# *trans*-Bis(2-acet­amido-5-methyl­benzoato-κ*O* ^1^)tetra­aqua­zinc

**DOI:** 10.1107/S1600536811012013

**Published:** 2011-04-07

**Authors:** Jin-Feng Huang, Jian-You Zheng, Yu-Mei Dai

**Affiliations:** a College of Chemistry & Material Science, Fujian Key Laboratory of Polymer Materials, Fujian Normal University, Fuzhou 350007, People’s Republic of China

## Abstract

In the title compound, [Zn(C_10_H_10_NO_3_)_2_(H_2_O)_4_], the Zn^II^ atom lies on a crystallographic inversion center and is six-coordinated by two monodentate *trans*-related 2-(*N*-acetyl­amino)-5-methyl­benzoato ligands and four water mol­ecules, giving a slightly distorted octa­hedral geometry. There are two intra­molecular hydrogen bonds [amine N—H⋯O_carbox­yl_ and water O—H⋯O_carbox­yl_], while extensive inter­molecular water O—H⋯O hydrogen-bonding inter­actions extend the complex units into a two-dimensional network structure along (100).

## Related literature

The study of metal coordination polymers has enhanced our understanding of the relationship between mol­ecular structure and material function, see: Dai *et al.* (2005 ▶); Moulton & Zaworotko (2001 ▶).
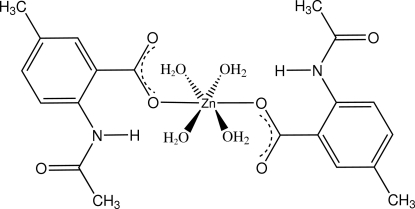


## Experimental

### 

#### Crystal data

[Zn(C_10_H_10_NO_3_)_2_(H_2_O)_4_]
*M* *_r_* = 521.83
Monoclinic, 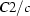

*a* = 19.300 (4) Å
*b* = 9.3000 (19) Å
*c* = 13.300 (3) Å
β = 107.60 (3)°
*V* = 2275.5 (9) Å^3^
*Z* = 4
Mo *K*α radiation
μ = 1.14 mm^−1^
*T* = 296 K
0.42 × 0.40 × 0.25 mm

#### Data collection

Bruker SMART CCD diffractometer
Absorption correction: multi-scan (*SADABS*; Sheldrick, 1996 ▶) *T*_min_ = 0.626, *T*_max_ = 0.752
10817 measured reflections
2130 independent reflections
1941 reflections with *I* > 2σ(*I*)
*R*_int_ = 0.029

#### Refinement

*R*[*F* ^2^ > 2σ(*F* ^2^)] = 0.055
*wR*(*F* ^2^) = 0.179
*S* = 1.11
2130 reflections
164 parameters
2 restraints
H atoms treated by a mixture of independent and constrained refinement
Δρ_max_ = 0.49 e Å^−3^
Δρ_min_ = −0.77 e Å^−3^

### 

Data collection: *SMART* (Bruker, 2004 ▶); cell refinement: *SAINT* (Bruker, 2004 ▶); data reduction: *SAINT*; program(s) used to solve structure: *SHELXS97* (Sheldrick, 2008 ▶); program(s) used to refine structure: *SHELXL97* (Sheldrick, 2008 ▶); molecular graphics: *SHELXTL* (Sheldrick, 2008 ▶); software used to prepare material for publication: *SHELXTL*.

## Supplementary Material

Crystal structure: contains datablocks I, global. DOI: 10.1107/S1600536811012013/zs2102sup1.cif

Structure factors: contains datablocks I. DOI: 10.1107/S1600536811012013/zs2102Isup2.hkl

Additional supplementary materials:  crystallographic information; 3D view; checkCIF report

## Figures and Tables

**Table 1:** Hydrogen-bond geometry (Å, °)

*D*—H⋯*A*	*D*—H	H⋯*A*	*D*⋯*A*	*D*—H⋯*A*
N1—H1*A*⋯O1	0.86	1.95	2.616 (4)	133
O1*W*—H1*WA*⋯O2^i^	0.81 (2)	1.90 (2)	2.704 (4)	170 (5)
O1*W*—H1*WB*⋯O3^ii^	0.83 (2)	1.88 (2)	2.707 (4)	177 (4)
O2*W*—H2*WA*⋯O2^i^	0.80 (4)	2.12 (4)	2.916 (5)	172 (5)
O2*W*—H2*WB*⋯O3^iii^	0.83 (4)	1.84 (3)	2.627 (4)	159 (5)

Symmetry codes: (i) 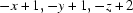; (ii) 
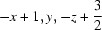
; (iii) 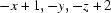.

**Fig. 1.**
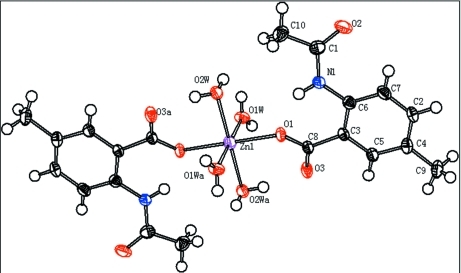
Local coordination around Zn ion in (I). Displacement ellipsoids are drawn at the 50% probability level. Symmetry code: (a): -x + 1, -y, -z + 2

**Fig. 2.**
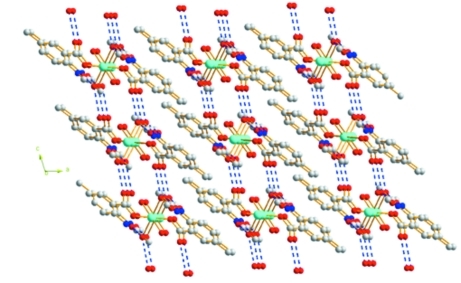
The crystal packing of (I) with hydrogen bonds shown as dashed lines.

## supplementary crystallographic information

### Comment

In recent decades, the study of metal coordination polymers has witnessed tremendous growth as an attractive interface between synthetic chemistry and materials science, which significantly boosts the understanding of the relationship between molecular structure and material function (Moulton & Zaworotko, 2001; Dai *et al.*, 2005). The crystal engineering of coordination polymers is highly influenced by the judicious choice of ligands, metal coordination geometry, template design and other subtle factors, such as counterions, solvent choice and reaction temperature. The deprotonated 2-(*N*-acetylamino)-5-methylbenzoic acid (HNB) ligands are good candidates in this respect for the construction of supramolecular architectures because in such bitopical ligands the *N*-acetyl group can act as a hydrogen-bond donor and/or acceptor, while the carboxyl function has strong coordination abilities with many metal ions. Taking these advantages into account, recently we have begun to assemble HNB and zinc ions into polymeric complexes under hydrothermal conditions. Herein, we report the synthesis and crystal structure of the title compound, [Zn(C_10_H_10_NO_3_)_2_(H_2_O)_4_] (I).

In the structure of (I) the Zn^II^ metal center lies on a crystallographic inversion center. The local coordination environment around Zn^II^ atom is slightly distorted octahedral, comprising two monodentate *trans*-related 2-(*N*-acetylamino)-5-methylbenzoato ligands and four water molecules (Fig. 1). Two intramolecular hydrogen bonds [amine N—H···O_carboxyl_ and water O—H···O_carboxyl_] stabilize the complex units while extensive intermolecular water O—H···O_acetyl_ hydrogen-bonding interactions are observed in the structure (Table 1), giving rise to double-stranded chains. Further interactions involving the coordinated water ligands and the uncoordinated O atoms of the carboxyl group are gives a two-dimensional network structure (Fig. 2).

### Experimental

ZnSO_4_^.^7H_2_O (1.00 mmol, 0.28 g), 2-(*N*-acetylamino)-5-methylbenzoic acid (HNB) (1.00 mmol, 0.19 g) and NaOH (1.00 mmol, 0.04 g) were mixed in water (15 ml) and heated at 403 K for 3 days in a sealed 25 ml Teflon-lined stainless steel vessel under autogenous pressure. After cooling to room temperature at a rate of 5° C h^-1^, yellow block crystals were isolated, washed with ethanol and then dried in air (33% yield).

### Refinement

H atoms attached to carbon and nitrogen were positioned geometrically and treated using a riding model, fixing the bond lengths at 0.86, 0.96 and 0.93 Å for NH, CH_2_ and aromatic CH groups, respectively and *U*_iso_(H) = 1.2*U*_eq_(N, C)]. The aqua H atoms were located from difference maps and their coordinates refined but with *U*_iso_(H) = 1.5*U*_eq_(O).

### Crystal data

**Table:**

[Zn(C_10_H_10_NO_3_)_2_(H_2_O)_4_]	*F*(000) = 1088
*M**_r_* = 521.83	*D*_x_ = 1.523 Mg m^−^^3^
Monoclinic, *C*2/*c*	Mo *K*α radiation, λ = 0.71073 Å
Hall symbol: -C 2yc	Cell parameters from 4107 reflections
*a* = 19.300 (4) Å	θ = 3.1–25.6°
*b* = 9.3000 (19) Å	µ = 1.14 mm^−^^1^
*c* = 13.300 (3) Å	*T* = 296 K
β = 107.60 (3)°	Block, yellow
*V* = 2275.5 (9) Å^3^	0.42 × 0.40 × 0.25 mm
*Z* = 4	

### Data collection

**Table:**

Bruker SMART CCD diffractometer	2130 independent reflections
Radiation source: fine-focus sealed tube	1941 reflections with *I* > 2σ(*I*)
graphite	*R*_int_ = 0.029
ω scans	θ_max_ = 25.6°, θ_min_ = 3.1°
Absorption correction: multi-scan (*SADABS*; Sheldrick, 1996)	*h* = −23→21
*T*_min_ = 0.626, *T*_max_ = 0.752	*k* = −10→11
10817 measured reflections	*l* = −16→16

### Refinement

**Table:**

Refinement on *F*^2^	Primary atom site location: structure-invariant direct methods
Least-squares matrix: full	Secondary atom site location: difference Fourier map
*R*[*F*^2^ > 2σ(*F*^2^)] = 0.055	Hydrogen site location: inferred from neighbouring sites
*wR*(*F*^2^) = 0.179	H atoms treated by a mixture of independent and constrained refinement
*S* = 1.11	*w* = 1/[σ^2^(*F*_o_^2^) + (0.1075*P*)^2^ + 9.1602*P*] where *P* = (*F*_o_^2^ + 2*F*_c_^2^)/3
2130 reflections	(Δ/σ)_max_ = 0.001
164 parameters	Δρ_max_ = 0.49 e Å^−^^3^
2 restraints	Δρ_min_ = −0.77 e Å^−^^3^

### Special details

**Table:**

Geometry. All e.s.d.'s (except the e.s.d. in the dihedral angle between two l.s. planes) are estimated using the full covariance matrix. The cell e.s.d.'s are taken into account individually in the estimation of e.s.d.'s in distances, angles and torsion angles; correlations between e.s.d.'s in cell parameters are only used when they are defined by crystal symmetry. An approximate (isotropic) treatment of cell e.s.d.'s is used for estimating e.s.d.'s involving l.s. planes.
Refinement. Refinement of *F*^2^ against ALL reflections. The weighted *R*-factor *wR* and goodness of fit *S* are based on *F*^2^, conventional *R*-factors *R* are based on *F*, with *F* set to zero for negative *F*^2^. The threshold expression of *F*^2^ > σ(*F*^2^) is used only for calculating *R*-factors(gt) *etc*. and is not relevant to the choice of reflections for refinement. *R*-factors based on *F*^2^ are statistically about twice as large as those based on *F*, and *R*- factors based on ALL data will be even larger.

### Fractional atomic coordinates and isotropic or equivalent isotropic displacement parameters (Å^2^)

**Table:**

	*x*	*y*	*z*	*U*_iso_*/*U*_eq_	
Zn1	0.5000	0.0000	1.0000	0.0283 (3)	
N1	0.61735 (18)	0.4086 (4)	1.0380 (3)	0.0268 (7)	
H1A	0.6034	0.3279	1.0569	0.032*	
O1W	0.42661 (16)	0.0994 (3)	0.8725 (2)	0.0280 (6)	
H1WA	0.410 (2)	0.173 (3)	0.890 (3)	0.034*	
H1WB	0.422 (3)	0.085 (4)	0.8095 (18)	0.034*	
O1	0.57941 (14)	0.1422 (3)	0.9864 (2)	0.0247 (6)	
O2W	0.47378 (16)	0.1539 (3)	1.1025 (2)	0.0299 (7)	
H2WA	0.449 (2)	0.211 (4)	1.063 (3)	0.036*	
H2WB	0.456 (2)	0.097 (4)	1.136 (3)	0.036*	
O2	0.6109 (2)	0.6481 (3)	1.0578 (3)	0.0428 (8)	
O3	0.59193 (17)	0.0609 (3)	0.8358 (2)	0.0328 (7)	
C1	0.5978 (2)	0.5248 (5)	1.0801 (3)	0.0296 (9)	
C2	0.7488 (2)	0.4914 (4)	0.8911 (4)	0.0296 (10)	
H2A	0.7806	0.5647	0.8871	0.035*	
C3	0.65394 (19)	0.2748 (4)	0.9062 (3)	0.0208 (7)	
C4	0.7454 (2)	0.3684 (4)	0.8315 (3)	0.0269 (8)	
C5	0.6976 (2)	0.2625 (4)	0.8402 (3)	0.0244 (8)	
H5A	0.6943	0.1792	0.8003	0.029*	
C6	0.6584 (2)	0.3999 (4)	0.9657 (3)	0.0245 (8)	
C7	0.7056 (3)	0.5073 (4)	0.9566 (4)	0.0298 (10)	
H7A	0.7085	0.5916	0.9952	0.036*	
C8	0.60481 (19)	0.1505 (4)	0.9089 (3)	0.0218 (8)	
C9	0.7926 (2)	0.3494 (5)	0.7609 (4)	0.0374 (10)	
H9A	0.7825	0.2581	0.7259	0.056*	
H9B	0.8428	0.3532	0.8024	0.056*	
H9C	0.7827	0.4249	0.7093	0.056*	
C10	0.5580 (3)	0.4978 (5)	1.1599 (4)	0.0379 (11)	
H10A	0.5459	0.5881	1.1854	0.057*	
H10B	0.5884	0.4433	1.2178	0.057*	
H10C	0.5142	0.4449	1.1273	0.057*	

### Atomic displacement parameters (Å^2^)

**Table:**

	*U*^11^	*U*^22^	*U*^33^	*U*^12^	*U*^13^	*U*^23^
Zn1	0.0351 (5)	0.0249 (4)	0.0276 (4)	−0.0034 (2)	0.0136 (3)	−0.0013 (2)
N1	0.0366 (18)	0.0198 (16)	0.0284 (17)	−0.0013 (14)	0.0166 (14)	−0.0030 (13)
O1W	0.0396 (16)	0.0238 (14)	0.0197 (13)	0.0070 (12)	0.0077 (12)	−0.0002 (11)
O1	0.0332 (14)	0.0225 (13)	0.0230 (13)	−0.0110 (11)	0.0154 (11)	−0.0047 (10)
O2W	0.0450 (17)	0.0217 (14)	0.0295 (15)	−0.0044 (12)	0.0210 (13)	−0.0029 (11)
O2	0.066 (2)	0.0219 (16)	0.0467 (19)	0.0081 (14)	0.0265 (17)	0.0020 (13)
O3	0.0511 (18)	0.0284 (15)	0.0262 (15)	−0.0162 (14)	0.0224 (13)	−0.0083 (12)
C1	0.030 (2)	0.031 (2)	0.026 (2)	0.0014 (17)	0.0064 (17)	−0.0044 (17)
C2	0.031 (2)	0.024 (2)	0.035 (2)	−0.0084 (15)	0.0119 (18)	0.0032 (15)
C3	0.0233 (17)	0.0192 (17)	0.0194 (17)	−0.0015 (14)	0.0058 (14)	0.0028 (14)
C4	0.0258 (19)	0.032 (2)	0.0234 (19)	−0.0044 (16)	0.0076 (15)	0.0052 (16)
C5	0.0286 (19)	0.0226 (18)	0.0230 (18)	−0.0039 (15)	0.0093 (15)	−0.0005 (14)
C6	0.0295 (19)	0.0223 (19)	0.0215 (18)	−0.0003 (16)	0.0076 (15)	0.0006 (15)
C7	0.038 (2)	0.020 (2)	0.033 (2)	−0.0051 (15)	0.012 (2)	−0.0021 (15)
C8	0.0249 (18)	0.0198 (17)	0.0215 (18)	−0.0028 (14)	0.0084 (15)	0.0023 (14)
C9	0.038 (2)	0.043 (3)	0.038 (2)	−0.0123 (19)	0.021 (2)	−0.0001 (19)
C10	0.043 (3)	0.039 (3)	0.037 (3)	−0.0061 (18)	0.021 (2)	−0.0118 (18)

### Geometric parameters (Å, °)

**Table:**

Zn1—O1W^i^	2.070 (3)	C2—C7	1.384 (7)
Zn1—O1W	2.070 (3)	C2—C4	1.382 (6)
Zn1—O1	2.073 (2)	C2—H2A	0.9300
Zn1—O1^i^	2.073 (2)	C3—C5	1.393 (5)
Zn1—O2W	2.140 (3)	C3—C6	1.395 (5)
Zn1—O2W^i^	2.140 (3)	C3—C8	1.503 (5)
N1—C1	1.324 (5)	C4—C5	1.379 (5)
N1—C6	1.421 (5)	C4—C9	1.503 (6)
N1—H1A	0.8600	C5—H5A	0.9300
O1W—H1WA	0.811 (18)	C6—C7	1.382 (6)
O1W—H1WB	0.826 (18)	C7—H7A	0.9300
O1—C8	1.270 (4)	C9—H9A	0.9600
O2W—H2WA	0.80 (4)	C9—H9B	0.9600
O2W—H2WB	0.83 (4)	C9—H9C	0.9600
O2—C1	1.230 (5)	C10—H10A	0.9600
O3—C8	1.247 (5)	C10—H10B	0.9600
C1—C10	1.508 (6)	C10—H10C	0.9600
			
O1W^i^—Zn1—O1W	180.00 (12)	C5—C3—C6	118.7 (3)
O1W^i^—Zn1—O1	90.89 (11)	C5—C3—C8	117.2 (3)
O1W—Zn1—O1	89.11 (11)	C6—C3—C8	124.0 (3)
O1W^i^—Zn1—O1^i^	89.11 (11)	C5—C4—C2	117.4 (4)
O1W—Zn1—O1^i^	90.89 (11)	C5—C4—C9	121.1 (4)
O1—Zn1—O1^i^	180.0	C2—C4—C9	121.5 (4)
O1W^i^—Zn1—O2W	90.67 (11)	C4—C5—C3	122.8 (4)
O1W—Zn1—O2W	89.33 (11)	C4—C5—H5A	118.6
O1—Zn1—O2W	87.28 (10)	C3—C5—H5A	118.6
O1^i^—Zn1—O2W	92.72 (10)	C7—C6—C3	118.8 (4)
O1W^i^—Zn1—O2W^i^	89.33 (11)	C7—C6—N1	122.4 (3)
O1W—Zn1—O2W^i^	90.67 (11)	C3—C6—N1	118.7 (3)
O1—Zn1—O2W^i^	92.72 (10)	C6—C7—C2	121.1 (4)
O1^i^—Zn1—O2W^i^	87.28 (10)	C6—C7—H7A	119.4
O2W—Zn1—O2W^i^	180.000 (1)	C2—C7—H7A	119.4
C1—N1—C6	128.4 (3)	O3—C8—O1	123.9 (3)
C1—N1—H1A	115.8	O3—C8—C3	118.3 (3)
C6—N1—H1A	115.8	O1—C8—C3	117.8 (3)
Zn1—O1W—H1WA	112 (3)	C4—C9—H9A	109.5
Zn1—O1W—H1WB	126 (3)	C4—C9—H9B	109.5
H1WA—O1W—H1WB	119 (3)	H9A—C9—H9B	109.5
C8—O1—Zn1	125.9 (2)	C4—C9—H9C	109.5
Zn1—O2W—H2WA	104 (4)	H9A—C9—H9C	109.5
Zn1—O2W—H2WB	98 (3)	H9B—C9—H9C	109.5
H2WA—O2W—H2WB	122 (3)	C1—C10—H10A	109.5
O2—C1—N1	123.5 (4)	C1—C10—H10B	109.5
O2—C1—C10	120.8 (4)	H10A—C10—H10B	109.5
N1—C1—C10	115.7 (4)	C1—C10—H10C	109.5
C7—C2—C4	121.1 (4)	H10A—C10—H10C	109.5
C7—C2—H2A	119.4	H10B—C10—H10C	109.5
C4—C2—H2A	119.4		

Symmetry codes: (i) −*x*+1, −*y*, −*z*+2.

### Hydrogen-bond geometry (Å, °)

**Table:**

*D*—H···*A*	*D*—H	H···*A*	*D*···*A*	*D*—H···*A*
N1—H1A···O1	0.86	1.95	2.616 (4)	133
O1W—H1WA···O2^ii^	0.81 (2)	1.90 (2)	2.704 (4)	170 (5)
O1W—H1WB···O3^iii^	0.83 (2)	1.88 (2)	2.707 (4)	177 (4)
O2W—H2WA···O2^ii^	0.80 (4)	2.12 (4)	2.916 (5)	172 (5)
O2W—H2WB···O3^i^	0.83 (4)	1.84 (3)	2.627 (4)	159 (5)

Symmetry codes: (ii) −*x*+1, −*y*+1, −*z*+2; (iii) −*x*+1, *y*, −*z*+3/2; (i) −*x*+1, −*y*, −*z*+2.

## References

[bb1] Bruker (2004). *SMART* and *SAINT* Bruker AXS Inc., Madison, Wisconsin, USA.

[bb2] Dai, Y. M., Ma, E., Tang, E., Zhang, J., Li, Z. J., Huang, X. & Yao, Y. G. (2005). *Cryst. Growth Des.* **5**, 1313–1315.

[bb3] Moulton, B. & Zaworotko, M. J. (2001). *Chem. Rev.* **101**, 1629–1639. 10.1021/cr9900432 11709994

[bb4] Sheldrick, G. M. (1996). *SADABS* University of Göttingen, Germany.

[bb5] Sheldrick, G. M. (2008). *Acta Cryst.* A**64**, 112–122. 10.1107/S0108767307043930 18156677

